# Low-Level Human Equivalent Gestational Lead Exposure Produces Supernormal Scotopic Electroretinograms, Increased Retinal Neurogenesis, and Decreased Retinal Dopamine Utilization in Rats

**DOI:** 10.1289/ehp.11268

**Published:** 2008-02-22

**Authors:** Donald A. Fox, Subbarao V. Kala, W. Ryan Hamilton, Jerry E. Johnson, James P. O’Callaghan

**Affiliations:** 1 College of Optometry; 2 Department of Biology and Biochemistry and; 3 Department of Pharmacology and Pharmaceutical Sciences, University of Houston, Houston, Texas, USA; 4 One Source Toxicology Laboratory, Inc., Pasadena, Texas, USA; 5 Department of Natural Sciences, University of Houston-Downtown, Houston, Texas, USA; 6 Toxicology and Molecular Biology Branch, Health Effects Research Laboratory, Centers for Disease Control and Prevention-National Institute of Occupational Safety and Health, Morgantown, West Virginia, USA

**Keywords:** bipolar cells, development, dopamine, electroretinograms, gestation, lead, neurogenesis, rod photoreceptors, scotopic, zinc

## Abstract

**Background:**

Postnatal lead exposure in children and animals produces alterations in the visual system primarily characterized by decreases in the rod-mediated (scotopic) electroretinogram (ERG) amplitude (subnormality). In contrast, low-level gestational Pb exposure (GLE) increases the amplitude of scotopic ERGs in children (supernormality).

**Objectives:**

The goal of this study was to establish a rat model of human equivalent GLE and to determine dose–response effects on scotopic ERGs and on retinal morphology, biochemistry, and dopamine metabolism in adult offspring.

**Methods:**

We exposed female Long-Evans hooded rats to water containing 0, 27 (low), 55 (moderate), or 109 (high) ppm of Pb beginning 2 weeks before mating, throughout gestation, and until postnatal day (PND) 10. We measured maternal and litter indices, blood Pb concentrations (BPb), retinal Pb concentrations, zinc concentrations, and body weights. On PND90, we performed the retinal experiments.

**Results:**

Peak BPb concentrations were < 1, 12, 24, and 46 μg/dL in control, low-, moderate- and high-level GLE groups, respectively, at PNDs 0–10. ERG supernormality and an increased rod photoreceptor and rod bipolar cell neurogenesis occurred with low- and moderate-level GLE. In contrast, high-level GLE produced ERG subnormality, rod cell loss, and decreased retinal Zn levels. GLE produced dose-dependent decreases in dopamine and its utilization.

**Conclusions:**

Low- and moderate-level GLE produced persistent scotopic ERG supernormality due to an increased neurogenesis of cells in the rod signaling pathway and/or decreased dopamine utilization, whereas high-level GLE produced rod-selective toxicity characterized by ERG subnormality. The ERG is a differential and noninvasive biomarker of GLE. The inverted U-shaped dose–response curves reveal the sensitivity and vulnerability of the developing retina to GLE.

The adverse effects of low-level developmental lead exposure [blood lead (BPb) ≤ 10 μg/dL] on cognitive, auditory, and visual-motor function are well documented (Canfield et al. 2003; Osman et al. 1999; Rothenberg et al. 2000; Wasserman et al. 2000), but only two studies have examined the impact of low-level developmental Pb exposure on retinal and visual function in children (Altmann et al. 1998; Rothenberg et al. 2002). This lack of research is despite findings that retinal and visual cortical structural–functional abnormalities occur in animals after moderate-level (BPb 11–39 μg/dL) and high-level (BPb ≥ 40 μg/dL) developmental Pb exposure (reviewed by Fox and Boyes 2007; Otto and Fox 1993).

Persistent rod photoreceptor-mediated (scotopic) electroretinographic (ERG) and behavioral deficits occur in monkeys, rats, and mice after moderate-level and high-level post-natal Pb exposure (PLE) (Fox and Boyes 2007). The scotopic ERG alterations are characterized by decreases in a-wave and b-wave ERG amplitude (subnormality), sensitivity, and temporal resolution. Similar ERG changes occurred in Pb-exposed workers and isolated retinas exposed to Pb^2+^ (Otto and Fox 1993). In contrast, adult monkeys with high-level lifetime Pb exposure exhibited increased ERG b-wave amplitudes (supernormality) (Lilienthal et al. 1994). Moreover, our prospective epidemiologic study of children with lifetime low-level and moderate-level Pb exposure revealed that only gestational Pb exposure (GLE) showed a significant dose-dependent relationship with supernormal ERG a-waves and b-waves and increased b-wave sensitivity with no change in implicit times (Rothenberg et al. 2002).

In experimental animals, supernormal scotopic ERGs are observed after the loss of retinal dopamine (DA) or zinc. For example, the destruction of dopaminergic amacrine cells by the neurotoxin 6-hydroxydopamine produced supernormal scotopic ERGs with normal implicit times (Olivier et al. 1986; Skrandies and Wässle 1988). Similarly, animals administered nonselective dopamine receptor antagonists had supernormal scotopic b-waves with normal implicit times (Jagadeesh et al. 1980; Schneider and Zrenner 1991). A persistent decrease in retinal tyrosine hydroxylase immunoreactivity after lifetime Pb exposure in monkeys suggests that a loss of DA produced supernormal ERGs (Kohler et al. 1997; Lilienthal et al. 1994). Chelation of retinal Zn produced supernormal scotopic ERGs and increased b-wave sensitivity (Redenti and Chappell 2003). Although no published reports have shown that scotopic ERG supernormality results from an increased number of rods, the log scotopic b-wave threshold and amplitude are linearly related to the number of rod photoreceptors and their rhodopsin content per eye (Dowling 1960). Therefore, we hypothesized that GLE produced supernormal scotopic ERGs by one of these three relatively independent mechanisms: an increased number of rod photoreceptors, decreased retinal DA metabolism, and/or decreased retinal Zn concentrations.

To determine the sites and mechanisms of the GLE-induced supernormal scotopic ERGs, we developed a new dose–response model of GLE. Then, we conducted scotopic ERG studies and retinal histologic, morphometric, and neurochemical experiments in adult offspring. Low-level and moderate-level GLE produced supernormal scotopic ERGs, an increased neurogenesis of rods and rod bipolar cells (BCs), and decreased DA synthesis and utilization/release in the absence of retinal injury. GLE produced inverted U-shaped dose–response curves as the high-level GLE dose produced rod cell loss and thus ERG subnormality.

## Materials and Methods

### Animals

All experimental and animal care procedures complied with the National Institutes of Health (NIH)/Public Health Service Policy on the Humane Care and Use of Laboratory Animals (NIH 2002) and were approved by the Institutional Animal Care and Use Committee of the University of Houston. All animals were treated humanely and with regard for alleviation of suffering. Adult (55–60 days of age) female and male Long-Evans hooded rats were obtained from Harlan Sprague Dawley, Inc. (Indianapolis, IN). The animals were housed in a room with a 12:12 hr light:dark cycle as described previously (Fox and Farber 1988).

We used two animal models: GLE and PLE. For each, dams were mated with a single male overnight, and the presence of a vaginal plug was recorded as gestational day 0.5. Control males were used once for breeding with Pb-exposed dams. Dams were weighed twice weekly until postnatal day (PND) 21 (weaning). On the day of birth (PND0), we recorded the number of pups, and sex and weight of the offspring (12–15 litters per group). On PND1, litters were culled to eight pups each. Only female rats were used to directly compare the present results with our previous ERG and retinal morphology studies conducted with adult female rats after PLE (reviewed by Fox and Boyes 2007). Pups were weighed on PNDs 7, 10, and 21. At weaning, rats were housed four per cage and weighed monthly. We performed ERG, morphologic, and neurochemical experiments at PND90. Protein values were obtained using the Bradford assay (Bradford 1976). For all analytical experiments, rats were decapitated 1–2 hr after light onset.

### GLE model

**Figure 1:**
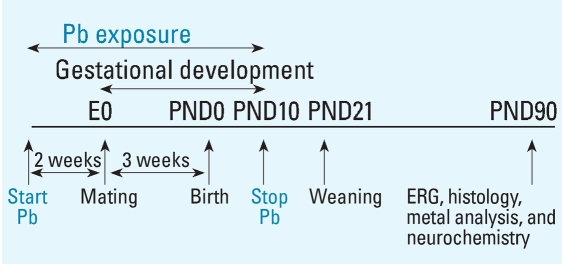
Rat model for human equivalent GLE. Female rats were exposed to Pb for 14 days before conception [embryonic day 0 (E0)] to establish steady-state BPb levels before mating. After mating, dams were exposed to Pb throughout gestation, and exposure was continued from birth (PND0) until PND10. This GLE model ensures that offspring were exposed for a period equivalent to the duration of human gestation. Experiments were conducted using adult (PND90) rats.

Two weeks after arrival, female rats were singly housed and randomly divided into four groups: one control group and three GLE groups. Control dams received water, and GLE dams received one of three Pb acetate drinking solutions (Fisher Scientific, Pittsburgh, PA): 0.005% (27 ppm Pb: low-level GLE), 0.01% (55 ppm Pb: moderate-level GLE), or 0.02% (109 ppm Pb: high-level GLE). Lead drinking solutions were provided to dams 2 weeks before mating to ensure BPb concentration stabilization and a Pb body burden, throughout gestation and until PND10. We selected the prenatal through PND10 period (Figure 1) because rodent brain and retinal development during this period is equivalent to that during human gestation (Dobbing and Sands 1979; Raedler and Sievers 1975; Rice and Barone 2000).

### PLE model

The details of this model are presented to distinguish the BPb concentration profiles and kinetics from those in GLE rats. Four weeks after arrival, female rats were singly housed and randomly divided into four experimental groups: one control and three PLE groups. Upon delivery and throughout lactation (PNDs 0–21), PLE dams received 0.005% (low-level PLE), 0.01% (moderate-level PLE), or a 0.02% (high-level PLE) Pb acetate drinking solution as previously described (Fox et al. 1991).

### Blood and retinal Pb, and retinal Zn concentrations

After decapitating the rats, we measured trunk BPb concentrations in GLE dams after 14 days of Pb pretreatment and on PND0. We measured trunk BPb concentrations in GLE offspring at PNDs 0, 10, 21, 30, and 90 and in PLE offspring at PNDs 10, 21, 45, 60, and 90. BPb concentrations, expressed as micrograms per deciliter, were measured by anodic stripping voltammetry using the LeadCare Kit1 (sensitivity: ≤ 1 μg/dL; Environmental Sciences Associates, Inc., Chelmsford, MA). Retinal Pb concentrations and Zn concentrations, expressed as parts per million (micrograms per gram wet weight), were measured in GLE offspring at PNDs 0, 10, 30, and 90 by anodic stripping voltam-metry or by atomic absorption spectrometry as previously described (Fox and Farber 1988). Values are reported for six to nine rats or five to seven retinas per age per group.

### Scotopic ERG procedures and analysis

ERG experiments were performed essentially as described by Fox and Farber (1988). We performed all procedures on dark-adapted rats under dim red illumination (λ > 650 nm). Rats were anesthetized with urethane (1.7 mg/kg, intraperitoneal injection), positioned in a Kopf stereotaxic apparatus, and placed on a heating pad that maintained core temperature at 37.0 ± 0.5°C. The left eye was covered with an occluder. The right cornea was anesthetized with 0.5% proparacaine hydrochloride, and the pupil was dilated with 1% atropine and 2.5% phenylephrine. A circular platinum–iridium recording electrode was positioned around the pupil, and platinum–iridium reference and ground electrodes were placed on the ear and tongue, respectively. Rats were then dark-adapted for 2 hr before ERG recordings.

Light stimulation was provided by a 300-watt quartz-iodine lamp through a Maxwellian optical system. The light was projected through a fiber optic cable that focused at the pupil and subtended 60° on the retina. Flash duration was 10 msec. ERG signals were amplified and filtered (bandwidth 0.1 Hz–1 KHz), displayed and monitored on an oscilloscope, and stored and analyzed on a computer. We recorded single-flash scotopic ERGs over a 7 log unit range of intensity [−4.5 to +2.5 log candela-seconds per square meter (cd-sec/m^2^)] in seven control rats.

In fully dark-adapted mammalian retinas, the single-photon rod signal is sequentially transmitted to depolarizing rod BCs, AII glycinergic amacrine cells, on and off cone BCs, on and off ganglion cells, and then to central visual centers (Bloomfield and Dacheux 2001). Rat rod BCs receive input from 16–25 rods (Greferath et al. 1990). The peak a-wave mostly reflects rod photocurrents but can have postreceptoral contributions (Robson et al. 2003), whereas the rat a-wave amplitude at 8 (Bui and Fortune 2004) or 10 msec (Mojumder and Frishman, personal communication) reflects rod photoreceptor function. We measured the negative-going a-wave amplitude at 10 msec after the flash and from baseline to its peak response. The peak of the positive-going b-wave amplitude, which reflects currents from depolarizing rod BCs and with additional BC-dependent K^+^ currents that affect Müller glial cells (Robson and Frishman 1999), was measured from the peak a-wave to peak b-wave, or in the absence of an a-wave from the baseline to its peak. We measured a-wave and b-wave peak implicit times. We fit b-wave amplitude data by the Michaelis-Menton equation using an iterative procedure that minimized the mean square error to ≤ 0.1%; flash intensities for b-wave half-saturation and maximal amplitude values were then determined (Rothenberg et al. 2002). Based on well-validated scotopic ERG testing procedures (Dalke et al. 2004), we used the flash intensities for the b-wave half-saturation value and maximal amplitude value as test stimulus 1 (low intensity) and test stimulus 2 (high intensity) to assess the effects of GLE on the scotopic ERG (*n* = 5–7 rats per GLE group).

### Epifluorescent and morphometric retinal studies

Rats were decapitated, and their eyes were rapidly removed and immersed in ice-cold phosphate-buffered saline (PBS), and the corneas were gently punctured. The eyes were immersion-fixed in 4% buffered para-formaldehyde for 30 min for epifluorescent studies or Karnovsky’s mixed-aldehyde fixative for 12 hr at 4°C for light microscopy as described previously (Fox and Chu 1988; He et al. 2000). For each experiment, we used five to seven retinas per GLE group.

For epifluorescent studies, fixed eyes were rinsed in PBS and cryoprotected. Anterior segments were removed, and eyecups were embedded in tissue-freezing medium and frozen in liquid nitrogen. Frozen retinas were sectioned along the vertical meridian (10–15 μm), collected on slides, and stored at –20°C. Sections were obtained from superior central retina (200–400 μm from the optic nerve head), labeled with the monoclonal mouse anti-protein kinase C (PKC)αβ clone MC5 antibody (BD Biosciences Pharmingen, San Jose, CA) that selectively stains rat rod BCs (Greferath et al. 1990; Osborne et al. 1992), and stained with AlexaFluor488-conjugated secondary antibody (Molecular Probes, Eugene, OR) or DAPI (4′,6-diamidino-2-phenylindole). We visualized and photographed retinas on an Olympus IX70 microscope (Olympus, Center Valley, PA) as previously described (Greferath et al. 1990; He et al. 2000).

We obtained counts of rod and cone photoreceptor cells (nuclei) in central and peripheral sections of the superior and inferior temporal retina of GLE rats from plastic sections (1–1.2 μm) stained with toluidine blue as described previously (Fox and Chu 1988; He et al. 2003). Nuclei counts were made from slides using a calibrated Filar micrometer eyepiece (Reichert Scientific Instruments, Buffalo, NY). We examined 20 fields, each 100 μm in length, in 3 sections per retina. Values are presented as rod and cone nuclei per 100 μm of retina because there were no significant differences between superior or inferior retina.

The outer nuclear layer (ONL), inner nuclear layer (INL), and total retinal thickness were measured from the same retinas (Fox and Rubinstein 1989). A change in retinal layer thickness reflects changes in cell number (Unoki and LaVail 1994). The ONL contains rods and cones, whereas the INL contains BCs, amacrine cells, horizontal cells, and Müller glial cells. We measured retinal layer and total retinal thickness in central superior and inferior retina with a Filar micrometer eyepiece 500 μm from the optic nerve head. Three repeat measures were obtained for each retina. Values for each GLE group are presented as retinal layer and total retinal thickness (micrometers) because there were no significant differences between superior or inferior retina.

### Rhodopsin measurements

GLE rats, dark-adapted overnight, were decapitated and the concentration of rhodopsin per eye was determined as described (Fox and Rubinstein 1989). The entire dark-adapted procedure was carried out under dim red light (λ > 650 nm). Visual pigment was extracted from two excised neuroretinas per rat with Emulphogene BC-720 (Gaf Corp., Wayne, NJ), and pre-bleach and postbleach scans were obtained from 350 to 700 nm with a Hewlett-Packard 8472A UV-VIS spectrophotometer (Hewlett-Packard, Palo Alto, CA). We obtained the absorbances of rhodopsin at its λ_maximum_ (497–500 nm) from difference spectra. Values are for five or six rats per GLE treatment group and are expressed as nanomoles per eye.

### Glial fibrillary acidic protein measurements

Glial fibrillary acidic protein (GFAP), the principal intermediate filament protein of glial cells, is a quantitative marker of glia; increased expression of GFAP is a marker of neuronal/glial damage or gliosis (O’Callaghan and Sriram 2005). We isolated and cleaned two retinas from each rat and determined retinal GFAP concentrations using the ELISA sandwich technique as described by O’Callaghan and Sriram (2005). Values are for three to five rats per GLE treatment group and are expressed as nanograms GFAP per milligram protein.

### Western blot analysis

To determine the concentration of PKCαβ and thereby the relative number of rod BCs, we conducted Western blotting experiments using the monoclonal mouse anti-PKCαβ clone MC5 antibody essentially as described previously (He et al. 2000; Osborne et al. 1992). Two retinas from each rat were isolated, cleaned in ice-cold PBS, and frozen at –80°C. Thawed retinas were homogenized in lysis buffer and centrifuged, and 20–30 μg protein was loaded onto a SDS-PAGE gel. Gels were stained with MC5 primary antibody and a goat anti-mouse IgG secondary antibody conjugated to horse-radish peroxidase (Jackson ImmunoResearch Laboratories, West Grove, PA), and visualized using an ECL Plus kit (Amersham Biosciences, Piscataway, NJ). We used a rabbit polyclonal GAPDH conjugated to horseradish peroxidase (ab9385; Abcam, Cambridge, MA), which yielded a 38-kDa band, as a loading control. We quantified the intensities of the bands using NIH Image 1.62 software. Values are for three to five rats per GLE treatment group.

### HPLC studies

Retinas were rapidly removed, cleaned, placed in ice-cold 0.2 N perchloric acid, and frozen at –80°C. Frozen samples were homogenized and centrifuged at 4°C. We measured concentrations of retinal DA, dihydroxyphenylacetic acid (DOPAC), and homovanillic acid (HVA) in the supernatant with an ESA HPLC-electrochemical detector system (ESA, Bedford, MA) (Kala and Jadhav 1995). Values are for five to six rats per GLE treatment group and are expressed as nanograms per milligram protein. We used DOPAC/DA and HVA/DA concentration ratios as measures of DA utilization and release (Boireau et al. 1990).

### Statistical analysis

Only one animal per litter or one retina per animal was used for any measure. We analyzed group data by a one-way analysis of variance (ANOVA), with or without repeated measures, followed by post hoc multiple comparisons using Tukey’s honestly significant difference test when significant main effects of Pb were found (KaleidaGraph; Synergy Software, Reading, PA). Data are presented as mean ± SE, and the difference from controls is regarded as significant if *p* < 0.05.

## Results

### Animal models and BPb, retinal Pb, and Zn levels

We measured fluid consumption and body weight of dams exposed to water or Pb from 14 days before conception until birth, and we recorded gestational and litter measures/milestones. Control values (Table 1) were similar to those previously reported for Long-Evans hooded rats (Church et al. 1990; Newland and Reile 1999). We found no significant differences between groups on any maternal or litter measure/milestone (data not shown). Table 1 presents the body weights of control rats at PNDs 0, 10, 21, and 90. There were no significant effects of GLE on body weight at any age (data not shown).

**Figure 2:**
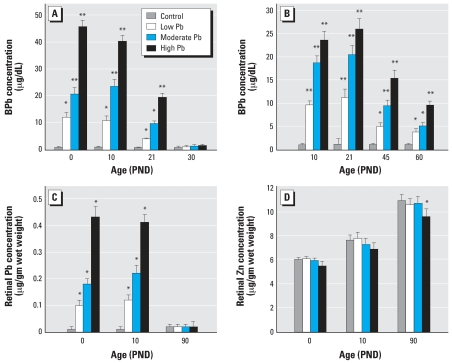
BPb, retinal Pb, and Zn concentrations. (*A*) BPb concentrations at PNDs 0, 10, and 21, but not PND30 were significantly different in GLE from controls. (*B*) BPb concentrations at PNDs 10, 21, 45, and 60 were significantly different in PLE from controls. (*C*) Retinal Pb concentrations at PNDs 0 and 10, but not PND30 were significantly different in GLE from controls. (*D*) Retinal Zn concentration at PND90 was significantly different in high-level GLE from controls. *p < 0.05, and **p < 0.01, compared with corresponding controls.

Figure 2A shows that control, low-level, moderate-level, and high-level GLE produced concentration-dependent increases in BPb concentrations at PNDs 0–10 with peak BPb concentrations of 1, 10–12, 21–24, and 40–46 μg/dL, respectively. By PND30, the BPb level in GLE rats was not significantly different from controls. Figure 2B shows that control, low-level, moderate-level, and high-level PLE produced significant concentration-dependent increases in BPb levels from PND10 to PND21, with peak BPb concentrations of 1, 11, 21, and 26 μg/dL, respectively, and that BPb concentrations were still significantly elevated at PND45 and PND60. By PND90, the BPb level in PLE rats was not significantly different from controls (data not shown) (Fox et al. 1991). Retinal Pb concentrations mirrored those of BPb for all GLE groups (Figure 2C) and PLE groups (Fox and Farber 1988; Fox et al. 1991; data not shown). Except for the high-level GLE, retinal Zn concentration was not significantly changed (Figure 2D).

### Single-flash scotopic ERGs

**Figure 3:**
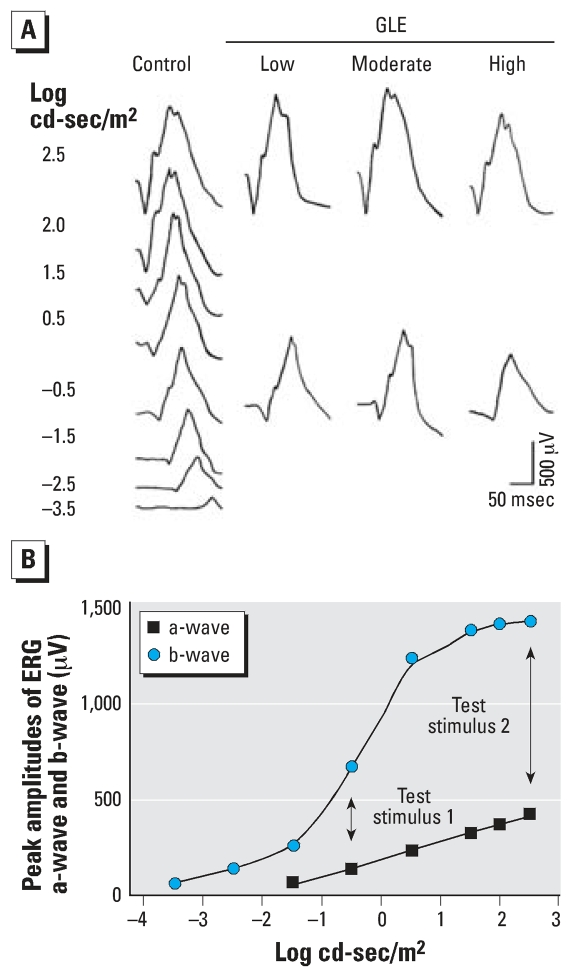
Single-flash scotopic ERG waveforms of adult control and GLE rats (*A*) and voltage-log intensity plot of ERG a- and b-waves for controls (*B*). (*A*) Single-flash scotopic ERGs were recorded over a 7 log unit range of intensity for controls and at a high (2.5 log cd-sec/m^2^) and low (−0.5 log cd-sec/m^2^) stimulus intensity in GLE rats. (*B*) Flash intensities for the b-wave half-saturation value (test stimulus 1: low intensity) and maximal amplitude value (test stimulus 2: high intensity) were used to assess the effects of GLE on scotopic ERGs.

Representative single-flash ERGs from fully dark-adapted adult control and GLE rats are illustrated in Figure 3A. The a-wave and b-wave voltage-log intensity curves for control rats (Figure 3B) show that the b-wave reached its maximum amplitude and plateaued at 2.5 log cd-sec/m^2^, whereas the a-wave had not plateaued. We used this stimulus intensity as test stimulus 2 (high intensity) in our ERG protocol. The stimulus intensity for the b-wave half-saturation value was –0.5 log cd-sec/m^2^, which was used as test stimulus 1 (low intensity) in our ERG protocol (Figure 3B).

**Figure 4:**
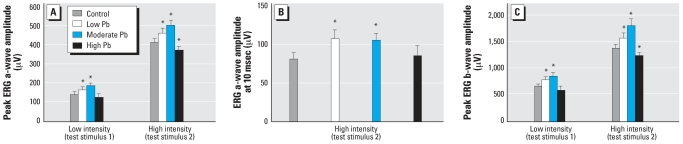
a-Wave and b-wave ERG amplitudes in adult control and GLE rats. (*A*) Relative to controls, peak ERG a-wave amplitude increased significantly in both low-level and moderate-level GLE groups, whereas it decreased significantly in high-level GLE rats with the high-intensity stimulus. (*B*) ERG a-wave amplitude at 10 msec increased significantly in both low-level and moderate-level GLE groups compared with controls. (*C*) Relative to controls, peak ERG b-wave amplitude increased significantly in low-level and moderate-level GLE groups, whereas it decreased significantly in high-level GLE rats with the high intensity stimulus. *p < 0.05, compared with corresponding controls.

In controls, peak a-wave amplitude with the low- and high-intensity stimuli were 141 ± 14 and 409 ± 19 μV, respectively (Figure 4A). The peak a-wave amplitude increased significantly in the low-level GLE (12–16%) and moderate-level GLE (22–26%) groups at both stimulus intensities, whereas it decreased significantly (11%) in the high-level GLE group at the high intensity stimulus (Figure 4A). In controls, a-wave amplitude at 10 msec was 79 μV ± 8.0 (Figure 4B). This increased significantly (30%) in the low-level and moderate-level GLE groups (Figure 4B). In controls, peak b-wave amplitude with the low- and high-intensity stimuli were 670 ± 28 and 1,387 ± 41 μV, respectively (Figure 4C). The peak b-wave amplitude increased significantly in the low-level (14–19%) and moderate-level (28–31%) GLE groups at both stimulus intensities, whereas it decreased significantly (10%) in the high-level GLE group at the high-intensity stimulus (Figure 4C). The increased peak b-wave amplitude at high stimulus intensity was significantly larger in the moderate-level than in the low-level GLE group. The control peak a-wave and b-wave latencies at the low (47.6 ± 4.1 and 92.2 ± 7.9, respectively) and high (20.7 ± 1.7 and 68.6 ± 5.8, respectively) stimulus intensities were not significantly different compared with those in the GLE groups.

### Retinal morphometry

**Figure 5:**
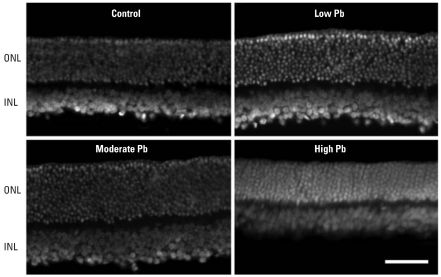
Histologic staining of adult control and GLE retinas with DAPI. Relative to controls, the thickness of the ONL and INL nuclear layers appears to be increased in low-level and moderate-level GLE rats, whereas the ONL thickness appears to be decreased in high-level GLE rats. Bar = 20 μm.

**Figure 6:**
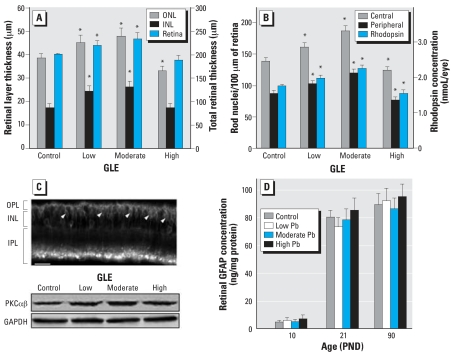
Morphometric and biochemical analysis of retinas from adult control and GLE rats. (*A*) Relative to controls, the ONL, INL, and total retinal thickness increased significantly in low-level and moderate-level GLE rats, indicating an increased neurogenesis, whereas the ONL thickness significantly decreased in high-level GLE rats. (*B*) Compared with controls, the number of ONL rod nuclei and retinal rhodopsin levels increased significantly in low-level and moderate-level GLE rats, whereas these measures decreased significantly in high-level GLE rats. (*C*) PKCαβ immunoblots suggest that PKCαβ-positive rod BCs (top) increased significantly in low-level and moderate-level GLE rats relative to controls, which is consistent with the INL data in (*A*); arrowheads indicate BC bodies. (*D*) Retinal GFAP was not affected by GLE exposure. *p < 0.05, compared with corresponding controls.

Representative images of DAPI-stained adult GLE retinas revealed that low-level and moderate-level GLE increased ONL and INL thickness (Figure 5). These increases reflect an increase of one to three nuclei per retinal layer. In controls, the ONL, INL, and total central retinal thickness was 38.7 ± 3.0, 17.5 ± 1.6, and 201.2 ± 2.1 μm, respectively (Figure 6A). Low-level and moderate-level GLE significantly increased ONL thickness (17–24%), INL thickness (40–50%), and total retinal thickness (10–16%), whereas high-level GLE significantly decreased ONL thickness by 14% (Figure 6A). In control central and peripheral retina, there were 137.8 ± 5.6 and 87.3 ± 4.3 rod nuclei per 100 μm, respectively (Figure 6B), and 2.9 ± 0.3 and 1.7 ± 0.3 cone nuclei per 100 μm, respectively. These values are consistent with our previous results (Fox and Chu 1988). Low-level and moderate-level GLE exhibited significant dose-dependent increases in the number of rods in central (16% and 35%, respectively) and peripheral retina (18% and 38%, respectively). In contrast, high-level GLE significantly decreased the number of rods in central and peripheral retina: 10% and 12%, respectively (Figure 6B). The number of cones was not changed significantly by GLE treatment.

We measured the level of rhodopsin per eye to determine whether the GLE-induced changes in rod nuclei density corresponded to a changed amount of the rod G-protein coupled receptor. In controls, the rhodopsin per eye was 1.97 ± 0.4 nmol (Figure 6B), consistent with our previous results (Fox and Rubinstein 1989). Low-level GLE (13%) and moderate-level GLE (29%) exhibited significant dose-dependent increases in rhodopsin concentration per eye, whereas it decreased significantly (12%) in high-level GLE rats (Figure 6B).

Morphometric analysis showed that low-level and moderate-level GLE increased INL thickness (Figure 6A). Examination of the retinas suggested that the number of BCs was increased by GLE, consistent with our supernormal b-wave results (Figure 4C). A control retina immunostained for PKCαβ showed that the entire rod BC selectively labeled with PKCαβ (Figure 6C), as described by Greferath et al. (1990). PKCαβ content significantly increased in the low-level (21%) and moderate-level (37%) GLE groups, consistent with the increase in INL thickness (Figures 5 and 6A), and was not significantly different in the high-level GLE group (Figure 6C).

### Retinal glial fibrillary acidic protein

In control retinas, GFAP expression increased from 5 ng/mg protein at PND10 to adult levels (80–90 ng/mg protein) at PND21 (Figure 6D). We found no significant effects of GLE on this measure at any age, indicating that there was no change in the number of Müller glial cells and no retinal injury.

### Retinal dopamine metabolism

**Figure 7:**
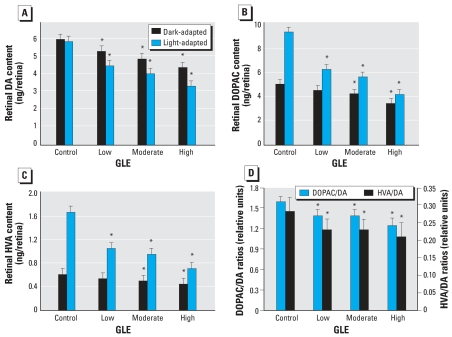
Retinal DA metabolism in adult control and GLE rats. GLE produced significant dose-dependent decreases in dark-adapted and light-adapted contrations of (*A*) DA, (*B*) DOPAC, and (*C*) HVA. Significantly larger changes occurred during light adaptation. (*D*) GLE produced significant dose-dependent decreases in light-adapted DA utilization/release. *p < 0.05, compared with corresponding controls.

Figure 7A shows that the dark-adapted and light-adapted DA concentrations are similar in controls: 6.02 ± 0.37 and 5.90 ± 0.33 ng/mg protein, respectively. This occurs because light activates tyrosine hydroxylase, the rate-limiting step, to synthesize DA (Iuvone et al. 1978). In contrast, Figure 7A shows that low-level, moderate-level, and high-level GLE produced significant dose-dependent decreases in dark-adapted DA (12, 19, and 27%) and light-adapted DA (23, 31, and 43%). In controls, the light-adapted compared to dark-adapted levels of DOPAC increased 86% (Figure 7B). Low-level, moderate-level, and high-level GLE produced significant dose-dependent decreases in dark-adapted DOPAC (10, 16, and 32%) and light-adapted DOPAC (39, 40, and 55%) (Figure 7B). Similar dose-dependent GLE effects were observed for HVA, the other major DA metabolite (Figure 7C). The GLE-induced decreases in DA, DOPAC, and HVA concentrations were significantly larger in the light, suggesting that GLE inhibited tyrosine hydroxylase and/or caused a loss of DA amacrine cells. Moreover, these GLE-induced changes in light-adapted DA metabolism resulted in dose-dependent decreases in DA utilization/release for DOPAC/DA and HVA/DA concentrations: 13–18% and 22–25%, respectively (Figure 7D).

## Discussion

In the present study, we obtained five novel results. First, we established a new and clinically relevant rat model of human equivalent GLE, an exposure period of increasing relevance and concern (Landrigan et al. 2005; Leasure et al. 2007; Rice and Barone 2000; Weiss et al. 2005). Second, low-level and moderate-level GLE produced supernormal scotopic ERGs in adult rats that are similar to our ERG findings in male and female children with GLE (Rothenberg et al. 2002). Third, low-level and moderate-level GLE increased neurogenesis of rod photoreceptors and rod BCs without affecting Müller glial cells. Fourth, GLE produced inverted U-shaped dose–response curves, as the high-level GLE produced subnormal ERGs and rod cell loss. Fifth, GLE produced dose-dependent decreases in adult retinal DA synthesis and utilization/release.

One of our most important and clinically relevant findings is that low-level and moderate-level GLE produced supernormal ERGs in adult rats. The BPb concentrations in these two groups are similar to those measured in pregnant women whose children had supernormal ERGs (Rothenberg et al. 2002). In contrast, high-level GLE produced subnormal ERGs similar to those observed with PLE (Fox and Farber 1988; Fox et al. 1991). These ERG results are characteristic of inverted U-shaped dose–response curves often observed in Pb neurotoxicity studies (Davis and Svendsgaard 1990; Leasure et al. 2007). These findings show that scotopic ERG is a sensitive, noninvasive biomarker that identified and discriminated low-level and moderate-level GLE from high-level GLE.

The GLE-induced increase in rods and rod BCs likely explains the presence of supernormal scotopic ERGs, as this would significantly amplify the rod signal. The molecular mechanism responsible for this novel retinal phenotype is unknown. Preliminary immuno-histochemical data indicate that the increase in rods and rod BCs is not due to a GLE-induced decrease in apoptosis during development. Thus, one attractive possibility is that a developmental decrease in retinal DA concentrations, similar to that observed in adult GLE offspring, mediated this increased neurogenesis. During early retinal development, DA influences proliferation and differentiation of progenitor cells by regulating the rate of mitosis (Kralj-Hans et al. 2006). Tyrosine hydroxylase–positive dopaminergic amacrine cells appear before PND0 and differentiate by PND14 (Wu and Cepko 1993)—the time period of GLE exposure and when the majority of late-born rods and BCs undergo terminal mitosis and differentiation (Rapaport et al. 2004). Thus, a decrease in retinal DA concentration during development would increase progenitor cell proliferation and produce more late-born rods and rod BCs.

High-level GLE also decreased retinal DA concentration. In contrast to the expected increase in late-born neurons and supernormal ERGs, high-level GLE offspring lost rods and had subnormal ERGs. These findings are similar to our retinal results with moderate-level and high-level PLE (Fox and Chu 1988; Fox and Farber 1988; Fox et al. 1991). Thus, our results reveal dose-dependent and developmental stage–dependent effects of Pb exposure. Moreover, the findings of decreased proliferation and/or neurogenesis in rat hippocampus after high-level GLE and/or PLE with peak BPb concentrations ≥ 60 μg/dl (Gilbert et al. 2005; Jaako-Movits et al. 2005; Verina et al. 2007) are consistent with our results and conclusions.

A second hypothesis suggests that low-to-moderate decreases in DA metabolism result in ERG supernormality, whereas larger decreases produce ERG subnormality. Scotopic ERG supernormality is a rare clinical finding; the only other reported occurrence is in drug-free patients with early Parkinson disease (Terziivanov et al. 1983). In advanced stages of Parkinson disease, the ERG is subnormal (Gottlob et al. 1987; Ikeda et al. 1994). This hypothesis is supported further by histologic, neurochemical, and ERG dose–response studies with 6-hydroxydopamine (Olivier et al. 1986; Skrandies and Wässle 1988) and our results. Together these findings indicate that the ERG reversed from supernormal to subnormal when retinal DA concntrations decreased > 40% and light-induced DA synthesis or utilization/release decreased > 20%. Although the mechanisms that underlie these ERG changes are unknown, they may involve the inhibitory effects of DA on rod Na^+^,K^+^-ATPase activity (Shulman and Fox 1996). Finally, decreases in retinal DA metabolism increase the risk of spatiotemporal contrast sensitivity deficits in adult humans and animals (Bodis-Wollner 1990), as observed in monkeys with lifetime Pb exposure (Rice 1998).

In summary, our results show that adult GLE rats with previous BPb concentrations of approximately 10 μg/dL, the current low-level of concern [Centers for Disease Control and Prevention (CDC) 1991], and approximately 20 μg/dL have persistent ERG supernormality, increased rods and rod BC neurogenesis, and decreased retinal DA synthesis and utilization/release. The nonmonotonic dose-dependent responses reveal that high-level GLE produced opposite morphologic and ERG effects. These data raise complex issues for risk assessment and indicate that dose-dependent and developmental stage-dependent effects are important components in risk assessment for neurotoxicity.

## Figures and Tables

**Table 1:** Maternal and litter measures for control Long-Evans hooded rats.

Measure	Outcome results
Maternal	
Dams’ daily mean fluid consumption (mL/day)
For 14 days before mating	14.7 ± 0.8
During gestation	29.5 ± 1.8
PND1–PND10	46.8 ± 3.7
Dams’ weight (g)
14 days before mating	223 ± 6
At mating	247 ± 7
Total weight gain during pregnancy	127 ± 6
Mating success rate (%)	93.8 ± 1.2
Litter and physical milestones	
Length of gestation (days)	20.5 ± 0.2
Mean litter size (pups)	11.9 ± 0.4
Sex distribution at birth [male/female (%)]	54/46
Litter mortality (dead newborn pups/litter)	0.2 ± 0.1
Pup mortality during lactation (%)	1.9 ± 0.1
Eye opening [male/female (%)]
At PND14, one or both eyes	78 ± 5/75 ± 6
At PND16, both eyes	100/100
Female offspring body weight (g)	
At birth (PND0)	6.12 ± 0.14
PND10	20.3 ± 1.2
PND21	50.1 ± 2.1
PND90	254.6 ± 4.8

Mean ± SE values are from 12–15 control dams and litters.
