# Erratum

**Published:** 2008-05

Fox et al. [ Environ Health Perspect 116:618–625 (2008) inadvertently used the wrong calibration unit in the text and Figure 3 of their article. Candela-seconds per square meter (cd-sec/m^2^) should have been Trolond-seconds (td-sec) throughout.

The authors regret the error.

