# Correction: Low-Level Human Equivalent Gestational Lead Exposure Produces Supernormal Scotopic Electroretinograms, Increased Retinal Neurogenesis, and Decreased Retinal Dopamine Utilization in Rats

**DOI:** 10.1289/ehp.11268-erratum

**Published:** 2008-06-01

Fox et al. [ Environ Health Perspect 116:618–625 (2008)] inadvertently used the wrong calibration unit in the text and Figure 3 of their article. Candela-seconds per square meter (cd-sec/m2) should have been Trolond-seconds (td-sec) throughout.

The authors regret the error.

